# FQI1: a transcription-methylation switch for cancer

**DOI:** 10.18632/oncotarget.15087

**Published:** 2017-02-04

**Authors:** Olga A. Guryanova, Jonathan D. Licht

**Affiliations:** Department of Medicine, Division of Hematology and Oncology, University of Florida College of Medicine, Gainesville, Florida, USA; Department of Biochemistry and Molecular Biology, University of Florida College of Medicine, Gainesville, Florida, USA

**Keywords:** oncogenesis, hepatocellular carcinoma, cancer epigenetics, LSF, DNA methylation

Rationally-designed therapies targeting oncogene addiction have shown significant clinical promise; however, resistance rapidly emerges, leading to disease relapse and treatment failure. Combination approaches aimed at both the oncogenic driver and a cooperating pathway are expected to deter development of resistance and prevent cancer recurrence. In this issue of Oncotarget Chin *et al*. describe FQI1, a small molecule inhibitor of the LSF-DNMT1 axis, that simultaneously targets a transcription factor oncogenic driver and an epigenetic modifier enforcing aberrant DNA methylation and altered gene expression.

Most hallmarks of cancer, whether sustained proliferation, resistance to apoptosis, or increased invasion, to name a few, ultimately stem from the acquisition of cancer-specific gene expression patterns [1]. In turn, gene expression is a sum of transcription factor binding and their ability to recruit epigenetic regulators. While mechanisms leading to altered gene expression can vary significantly, cancers often become uniquely dependent on a single activated oncogenic protein or signaling pathway, a concept termed oncogene addiction [2]. Acute inhibition of the oncogene culprit promptly leads to apoptosis and retarded proliferation, while non-transformed cells remain minimally affected. The resulting rapid tumor regression underlies the impressive clinical success of targeted therapies.

LSF is a grainyhead-related transcription factor that is highly expressed in hepatocellular carcinoma (HCC) and other malignancies. LSF overexpression is oncogenic in animal models, while its depletion promotes tumor shrinkage, making it an ideal anti-cancer target. Indeed, the Hansen group previously showed that FQI1, a small molecule, disrupted LSF DNA binding and suppressed LSF-driven transcription. This correlated with antiproliferative activity *in vitro* and rapid tumor regression of xenograft HCCs in mice [3]. Chin *et al*. now show that FQI1 can simultaneously target DNA methylation. The two-pronged mechanism of action makes FQI1 a promising drug candidate for further development.

Altered DNA methylation states are characteristic of cancer. DNA methylation patterns are established by the *de novo* DNA methyltransferases (DNMT) 3A and 3B, and copied to the daughter DNA strand by DNMT1 for maintenance during replication, although DNMT1 can also have a role in *de novo* DNA methylation [4]. Aberrant DNA methylation is a hallmark of cancer. Generally, tumor cells show global hypomethylation with focal DNA hypermethylation in tumor suppressor loci [5]. Consequently, reactivation of silenced tumor suppressor genes has been posited as one rationale for the clinical efficacy of hypomethylating agents, although recent studies suggest other effects such as de-repression of endogenous retroviruses, may also be at play [6]. DNMT inhibitors are a blunt instrument for reshaping DNA methylation in malignancy. Reactivation of just a subset of relevant genes might offer similar efficacy while avoiding undesirable side effects. Targeting the DNA methylation through the LSF-DNMT1 axis in HCC by FQI1 is an excellent example of such approach.

While gene activation by LSF has been well studied [7], its ability to repress genes was incompletely understood yet recognized to be important for its oncogenic activity. Chin *et al*. established that LSF binds to and stimulates DNMT1 activity to enforce methylation at specific loci. By disrupting the LSF-DNMT1 complex FQI1 impairs the genomic localization of DNMT1. The authors used reduced representation bisulfite sequencing (RRBS) to characterize ensuing genome-wide DNA methylation changes. Despite modest overall DNA hypomethylation, an almost equal number of hypermethylated differentially methylated regions (DMRs) was identified. Gene expression profiling by RNA-sequencing uncovered concordant changes in gene expression, as many DMRs were localized in gene promoter regions. Genes with increased expression upon FQI1 treatment included many implicated in cancer pathways, while inhibited genes were involved in DNA replication and cell cycle control. Interestingly, loci that underwent transcriptional activation also had higher levels of activating histone marks in steady state, suggesting that deregulation of these genes may be critical for the LSF-DNMT1 oncogenic function.

In addition to delineating a new epigenetic mechanism in cancer and a novel targeted therapeutic approach, this study raises several important questions. What mediates emergence of hypermethylated DNA loci after FQI1 treatment - are they due to delocalization and redistribution of DNMT1 that is no longer recruited by LSF? Are other DNMTs involved? Do changes in DNA methylation upon FQI1 exposure track together with histone marks, and if so, what is the timing of their deposition? Are there other oncogenic transcription factor-epigenetic modifier tandems that can be targeted in a similar manner? And finally, Chin et al. show that FQI1-mediated disruption of the LSF-DNMT1 axis results in altered expression of DNA replication genes. Since DNMT1 is loaded directly to the replication forks during the S-phase [4], would FQI1 further synergize with replication stress-inducing chemotherapy? Detailed profiling of the histone marks and DNMT1-bound loci by ChIP, in addition to evaluating rational combination treatment approaches will be eagerly awaited.

The discovery of a small molecule FQI1 that simultaneously targets two cooperating pathways in human cells improves our understanding of oncogene addiction mechanisms, and offers promise for the development of more specific and less toxic therapeutic approaches to improve outcomes for cancer patients.
